# A survey on herpes simplex type 2 antibody among pregnant women in Isfahan, Iran

**Published:** 2010

**Authors:** Azar Danesh Shahraki, Sharareh Moghim, Peyman Akbari

**Affiliations:** a Associate Professor of Obstetrics and Gynecology, Isfahan University of Medical Sciences, Isfahan, Iran; b Assistant Professor of Virology, Isfahan University of Medical Sciences, Isfahan, Iran; c General Practitioner, Isfahan University of Medical Sciences, Isfahan, Iran

Herpes simplex type 2 virus (HSV-2) infection is considered as the major cause of genital herpes. The recurrent infections are common but frequently asymptomatic.^1^ The virus could be transmitted vertically to the neonates from the mother shedding the virus during delivery, which could cause neonatal herpes in approximately 70% of neonates.^2^ Neonatal HSV-2 can have severe consequences and most surviving infants have neurological squeals.^3^ Lack of enough epidemiological data in our region in this field, made us to determine the frequency of HSV-2 infection among pregnant women in Isfahan. In this descriptive cross-sectional study, 96 pregnant women, attending gynecology clinic were studied. The overall prevalence of HSV-2 IgG was 42.96 (43.75%), assessed by ELISA kit (Captia^TM^ HSV2 IgG, Trinity biotech, with the sensitivity and specificity of 100% and 98.1%, respectively). The type cross reactivity relative to western blot has been determined 8%. HSV-2 infection is more prevalent in our region than western countries (32.4%) and less than turkey (53.3%).^4^,^5^ There was no significant relation between history of oral herpetic lesions, oral sexual habit and HSV-2 IgG in our study. There was significant relation between history of other genital lesions and HSV-2 IgG (p = 0.03). In conclusion, the occurrence of HSV-2 infection in our study was high and high proportion of our pregnant women are infected with the virus, which might be rarely serious in mothers but has severe consequences on neonates which indicates educating our population about its risk factors and awareness of the infection is necessary.
